# Engaging families in physical activity research: a family-based focus group study

**DOI:** 10.1186/s12889-015-2497-4

**Published:** 2015-11-25

**Authors:** Helen Elizabeth Brown, Annie Schiff, Esther M. F. van Sluijs

**Affiliations:** MRC Epidemiology Unit and UKCRC Centre for Diet and Activity Research (CEDAR), University of Cambridge School of Clinical Medicine, Institute of Metabolic Science, Box 285, Cambridge, CB2 0QQ UK

## Abstract

**Background:**

Family-based interventions present a much-needed opportunity to increase children’s physical activity levels. However, little is known about how best to engage parents and their children in physical activity research. This study aimed to engage with the *whole family* to understand how best to recruit for, and retain participation in, physical activity research.

**Methods:**

Families (including a ‘target’ child aged between 8 and 11 years, their parents, siblings, and others) were recruited through schools and community groups. Focus groups were conducted using a semi-structured approach (informed by a pilot session). Families were asked to order cards listing the possible benefits of, and the barriers to, being involved in physical activity research and other health promotion activities, highlighting the items they consider most relevant, and suggesting additional items. Duplicate content analysis was used to identify transcript themes and develop a coding frame.

**Results:**

Eighty-two participants from 17 families participated, including 17 ‘target’ children (mean age 9.3 ± 1.1 years, 61.1 % female), 32 other children and 33 adults (including parents, grandparents, and older siblings). Social, health and educational benefits were cited as being key incentives for involvement in physical activity research, with emphasis on children experiencing new things, developing character, and increasing social contact (particularly for shy children). Children’s enjoyment was also given priority. The provision of child care or financial reward was not considered sufficiently appealing. Increased time commitment or scheduling difficulties were quoted as the most pertinent barriers to involvement (especially for families with several children), but parents commented these could be overcome if the potential value for children was clear.

**Conclusions:**

Lessons learned from this work may contribute to the development of effective recruitment and retention strategies for children and their families. Making the wide range of potential benefits clear to families, providing regular feedback, and carefully considering family structure, may prove useful in achieving desired research participation. This may subsequently assist in engaging families in interventions to increase physical activity in children.

## Background

Family-based interventions present a much-needed opportunity to increase children’s physical activity levels. Parental support has been consistently and positively associated with increased physical activity in children [1], and the addition of parent education to school-based physical activity interventions has shown to be effective [2].

However, little is known about how best to engage parents and their children in physical activity promotion [3]. Recruitment rates are often not reported; a review of 23 family-based physical activity interventions was not able to include any information on recruitment compared with those eligible or invited to participate [4]. Failure to meet recruitment targets has several consequences. First, it may lead to underpowered research studies, where clinically relevant changes may appear statistically non-significant. Second, inadequate recruitment strategies may necessitate an extension of the recruitment period; which may be economically and logistically challenging. Third, it may result in recruitment of families which do not accurately represent the wider population, resulting in selection bias. Retention of families is equally difficult; in particular, children from socio-economically disadvantaged families, ethnic minorities, and those at risk of ill health, tend to drop out early [5]. The mean attrition rate (i.e., failure to complete measurement) reported in a review of child health studies was 20 % (range 0–54 %) at initial follow-up, and 32 % (range 0.59 %) for extended follow-up [5]. High attrition rates may also increase sampling bias and compromise generalizability [6].

Recent work consisting of a literature search and Delphi study presented potentially successful strategies for recruitment and retention of children in behavioural health risk factor studies [6]. Beneficial strategies reported included identifying suitable settings and tools for recruitment, eliciting support from key stakeholders/project champions, and ‘creating a study identity’. The authors of this work acknowledge, however, that their results are mainly driven by school-based intervention protocols, and may not be applicable for research conducted in other settings.

Family-focused work to date has engaged mostly with parents; soliciting their views on recruitment, content and delivery of family-based PA interventions [7–9], but none have involved all family members. For example, formative work for a recent family-based physical activity intervention included asking parents where best to advertise (schools were most commonly cited as a preferred locale for recruitment into research studies) [8] . A second study employed participatory processes to ensure parents were involved in all aspects of the intervention, including development of materials, and implementation of intervention activities [9]. However, neither of these involved *other* family members. To include parents, siblings, other family members, and the target children themselves, would be a novel way to explore recruitment and retention strategies. This inclusive strategy will enable participants to build on each other’s responses, and may help us to understand how family relationships facilitate or obstruct family physical activity.

The aim of this study was therefore to engage with the *whole family* to understand how best to recruit for, and retain participation in, family-based interventions to increase physical activity in children.

## Methods

### Ethics

Ethical approval for this study was granted by the Cambridge Psychology Research Ethics Committee (University of Cambridge, Application No: Pre.2013.119). All subsequent study amendments were reviewed and approved. Written (parental) consent was obtained for all participants, and child participants provided written assent.

### Pilot

A pilot focus group, using a convenience sample, was conducted to refine the study protocol. All procedures from recruitment through to data analysis were tested; pilot participants provided feedback, which was then used to improve each process. Data obtained in the pilot focus group was not included in the final study sample.

### Recruitment

Families (including a ‘target’ child aged between 8 and 11 years, their primary caregiver(s) (hereafter referred to as ‘parents’), siblings, and others involved in their care) were recruited through schools and community groups (e.g., scout/guide groups) in the wider Cambridgeshire area. Particular effort was made to contact schools and community groups (through which to deliver recruitment materials) that were located in areas representing all levels of socio-economic status. Initial letters and posters invited families to contact the study team to express their interest in participation. The study co-ordinator spoke with parents on the telephone, answered any questions, and obtained a postal and/or email addresses for further correspondence. Recruitment packets were then sent to families via their preferred method (i.e., postal or email), containing (i) a personalised cover letter, (ii) full study information with a list of frequently asked questions, (iii) written consent forms, and (iv) a brief data collection form. Personalised letters have been shown to elicit greater initial response [10], and therefore children’s, parent’s and sibling’s names were included in all correspondence. Once consent had been provided, appointments were scheduled to include all family members.

### Data collection procedures

Basic demographic and anthropometric data (age, sex, height, and weight) of those agreeing to participate were collected using the brief data collection form.

Focus groups were conducted in participants’ homes, mostly during the evening (to ensure all family members were available to participate). A semi-structured interview approach was used. An introductory activity was used as an ‘ice-breaker; participants were asked to share their favourite physical and non-physical activities. This encouraged each family member, along with researchers, to build rapport and ensured participants felt comfortable contributing to the discussion.

Families were then asked to order cards listing the possible benefits of, and the barriers to, being involved in physical activity research. These cards were used as prompts only (developed using feedback from our pilot focus group), and were not designed to provide an exhaustive list. They were asked to highlight the items they consider most relevant, and suggest additional items. Health, social, and educational benefits were listed, as well as the provision of child care, connection with other families, and incentives. Barriers included increased time commitment and/or scheduling clashes, difficulties with transport, financial commitment, and a lack of interest or perceived benefit. Participants were asked to explain their choices (i.e., items highlighted as being of most importance), discuss conflicting opinions, and supplement the list with additional items relevant to their family. Follow-up probes were used by the facilitator (HEB) to encourage further discussion. A second researcher (AS) recorded participant responses (particularly when ordering the cards) and noted family interactions. At the end of the session, AS provided families with a summary of the discussion, offering them a chance to elaborate or add to previous answers. Two digital voice recorders were used to record the session.

### Follow-up

All individual participants were provided with a £5 gift voucher and were contacted later with individual feedback on their session, and some interim results.

### Data analysis

Demographic data were recorded from the participant questionnaires. The postcode of each family home was used to classify according to socio-economic deprivation. Families were ranked according to social deprivation percentile (those ranked in the higher percentiles represent families of higher socio-economic status compared with the rest of the UK) [11].

Interviews were transcribed verbatim and anonymised by an external data transcription service. Data was then analysed thematically; two members of the study team (HEB and AS) independently read transcripts to identify emerging themes and develop a coding framework [12]. Once complete, the coding frame was provisionally tested with a 10 % sample of transcripts. Discrepancies were discussed, and the coding frame refined (on the basis of both theoretical issues guiding the research questions, and salient issues arising from the transcripts) [12]. Quotations were then clustered around broader themes and these themes were merged or altered where appropriate. Additionally, observer notes (recorded during the focus groups) were coded, and a quantitative count of the ‘card order’ (i.e., the order in which facilitators and barriers were listed by participants) was noted.

All data from the focus groups was recorded and considered, but the main aim of the present analysis was to identify effective recruitment and retention strategies for engaging families in physical activity interventions. Given the complexity of family context and relationships, family case studies were also written (combining demographic information, coded interview transcripts, and observer notes). This more detailed narrative aimed to offer further insight into the potential incentives for and barriers to research participation.

## Results

**Fig. 1:**
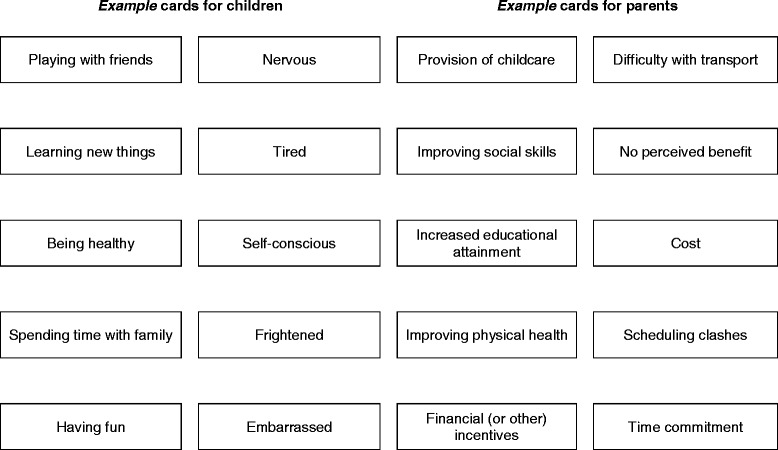
Examples of flashcards used to prompt facilitators/barriers discussion

**Fig. 2:**
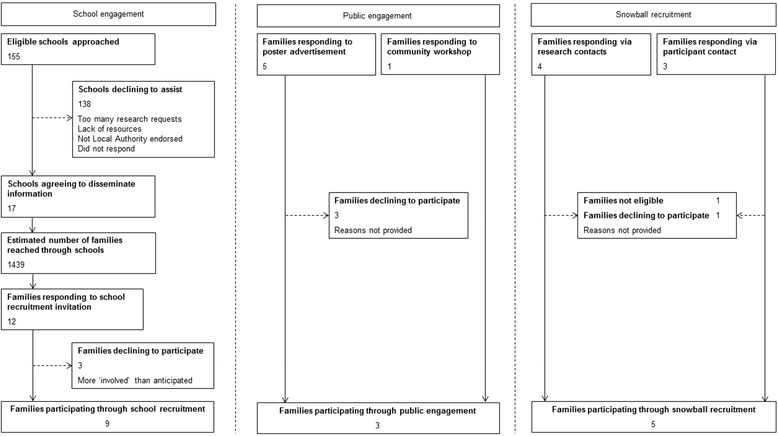
Recruitment of participants to family focus groups

Recruitment of participants to the study is depicted in Fig. 1. A total of 82 individual participants from 17 families took part (not including the pilot session). This included 17 ‘target’ children (mean age 9.3 ± 1.1 years, 61.1 % female), 32 other children (siblings) and 33 adults (including parents, grandparents, and older siblings). The median number of participants per focus group was 4 family members (range: 2–5). Self-identified ‘primary caregivers’ also provided demographic information; indicating a mean age of 44.2 ± 3.9 years. These respondents were mostly women (83.0 %). Socio-economic status (assessed from postcode) was relatively high; 89 % of families (15 families) were classified as being above the 70th percentile (and therefore of higher socio-economic status than the national average). The remaining two families were however, significantly lower; classified as above the 35th percentile, and above the 40th (Fig. 2).

### Incentives for involvement in research

Social benefits were cited as being key incentives for involvement in physical activity research, with emphasis on children experiencing new things (even “*being outdoors” (*grandmother: boy, 10y) was considered beneficial), developing character, and increasing social contact. This was particularly important for parents of shy children;

Mother: boy, 9y: *I think improved social skills is good, especially for shy children, I know he didn’t seem it but he can be shy sometimes so that’s quite nice to get them active and sort of connect to other people*

Mother: boy, 8y: *I mean the biggest thing I hope he improves is his social skills…communication with other kids…will get rid of [his] self-consciousness*

Achieving “*a balanced life*” (mother: boy, 10y) was also important, in which children were able to appreciate a variety of different activities. Making a connection, and “*fit[ting] in”* (father: girl, 11y), with the wider community was also cited as being of value. For most parents, research participation being of educational benefit for their children was essential (particularly, the children finding an activity “*stimulating*” (mother: boy 10y));

Mother: girls, 9y and 7y*: Well it makes them understand so I think that’s quite important, you know, like doing this* [participating in the present study], *it makes them understand why things happen so it kind of increases their educational attainment*

Fun was a key consideration for both parents and children;

Mother: boy, 10y: *But if there were things where it’s possible to go and enjoy it and … but if there were activities at the weekend that I thought were brilliantly pitched at their level, they would have fun, they’d learn, you know, new bowling skills and it didn’t matter if they didn’t go every week than I would be much more inclined to do it*

Mother: boy, 10y: O*ur priority would be, ‘is it something that I genuinely think they’d enjoy?’*

Families also gave priority to the perceived health outcomes of being involved in physical activity research, commenting that to be *“fitter…and possibly just getting stronger” (*grandmother: boy, 10y) would be of value. When asking the children what they considered to be the potential advantage of such research participation, weight loss or maintenance was commonly suggested;

Boy, 7y: T*he more active you are, the more weight you lose*

Girl, 9y: [You would take part in physical activity research] *because you don’t want to be fat!*

For most multiple-parent families (or families where practical support was given by other relatives), the provision of child care was not considered to be of sufficient appeal to encourage study participation, viewed as a “*useful”* (mother: girl, 11y) adjunct or bonus;

Mother: boy, 10y: *But it’s only childcare if it comes with some other things as well so it’s not just any old childcare*

Single parents, however, saw the provision of child care as more beneficial;

Mother: boy, 9y: *Providing childcare would be relevant to me ‘cos, as you know, I’m on my own*

Financial incentives for participation in research were not highly valued; families commented that the opportunity to contribute to something “*meaningful”* was of greater worth (mother: boy, 10y). Indeed, three families declined the offer of vouchers for involvement in the focus groups themselves. The receipt of detailed feedback was suggested as an alternative impetus;

Father: boy, 10y: B*eing informed of outcomes, and seeing progress in your child*

## Box 1 The Robinson family

**Table:** 

The Robinson family comprised a mother, father, and two children (James, aged 8, and Natalie, aged 14), and were of a higher socio-economic status than the study average. Their house was located in a quiet village in Cambridgeshire, with a large garden. Both parents were highly educated and very engaged, and were concerned with the educational and social development of James and Natalie. Character-building and personal achievement were frequently cited as important family values. Both Mr. and Mrs. Robinson understood the need to evaluate newly-designed interventions, and agreed that they would encourage James’ involvement if the benefits to his social development (learning to be calm, and patient) were clear. They were also motivated by health and educational outcomes. *Mr. Robinson: … I mean social skills is the sort of thing that Mrs. Robinson was talking about earlier on that sports give you generally and you know, as I said, I’m very sporty so I’ve enjoyed team games, I enjoy individual games and I see the benefits of both of those two, character development and all the rest of it … might be a particular benefit that I would see [for] James.* Mrs. Robinson, who spoke English as a second language, was also interested in research participation as a way of meeting and connecting with other families. *Mrs. Robinson: Provide other connections, that’s important for me because I think I quite enjoy meeting other people and know other people’s life and for him [James] to meet a different kind of group of people.* Mr. Robinson built on these comments, suggesting that participating with children he considered appropriate was also important. He suggested this may be related to class; and that the opportunity for James to interact with children who were well behaved, used suitable language, and provided positive role models, was appealing. *Mr. Robinson: The mixture of kids that he’d be with you know, … he plays tennis with lots of other middle-class kids who play tennis … they have a certain range in common … it’s also about a behaviour thing so giving you a specific example, he plays cricket at school, there’s a kid who is totally disruptive and just destroys the games, the teacher seems unable to deal with it to the point where effectively the parents have started withdrawing the kids from that activity and it’s just about that child’s behaviour and how the parents control that child’s behaviour, the language he uses and all that…*
*Mrs. Robinson: And I think if you take James to a group of new people he swears he wouldn’t want to go.* Both parents did not see financial reimbursement as an incentive for involvement in research studies. In fact, Mrs. Robinson declined the offer of vouchers as a thank you for their participation. James was most concerned with improving his skills, and cited learning new things as his primary motivation for being involved in research. This was a surprise to Natalie, who thought he would prioritise having fun. James also echoed his parents’ emphasis on achievement. He discussed his enjoyment of winning, and described himself as a ‘bad loser’. James and his family repeatedly commented on his competitive nature, and how much he enjoys playing against his friends. *James: Winning!* *… Facilitator: Yeah, it sounds like competition is really important for you.* *Mrs. Robinson: Yeah, he has that drive…* *Mr. Robinson: He’s pretty competitive.* *Mrs. Robinson: Yeah, he enjoys winning.* When discussing barriers to research participation, James talked about being embarrassed, and concerned about his friends’ reaction. Natalie indicated that he finds some activities ‘too girly’, and therefore would avoid studies featuring such activities. His parents suggested James’ fear of failure or losing may also be relevant. *Mr. Robinson: Mm, fear of failure.* *Natalie: Yeah.* *… Mr. Robinson: And it’s the flipside of the competitiveness and things and we’ve talked about it often with him that, you know, we’ll go along to something and he’ll say, well I’m going to get beaten by everybody, so how on earth can you know that, you haven’t played this before and so and so.*
*Facilitator: Okay, yeah.* *Mrs. Robinson: Oh yeah, yeah, and there’s no rational basis to it, it’s just kind of… But it is I think the flipside of that, being very keen on winning and also therefore not wanting to come anywhere other than, nothing less than the top three is allowed basically [Laughs].*

## Box 2 The Jones family

**Table:** 

The Jones family comprised a mother, father, and two children (Sophia, aged 5 and George, aged 10). Both parents were employed as university academics, and were actively engaged in the discussion. The children were articulate, open and keen to contribute to the conversation. At the close of the session, Mr. Jones and the children walked us to their local park, to demonstrate an area frequently used for ‘free play’. The family felt unstructured activity was important to children’s health and social development, and as such, outdoor space could be an appropriate setting for research. They enjoyed active holidays together (and being in nature); and regularly go trekking, camping, and skiing as a family. Given their profession, it was unsurprising that both parents were aware of, and committed to, the benefits of research participation. *Mr. Jones: … because there is no question that if the programme is well done there is … obvious benefit.* They listed health, educational and social outcomes are key drivers for engagement in physical activity studies. Mr. Jones was particularly keen that the children learn new things, and are offered a range of experiences. *Mr. Jones: … you know, for George to see new things, learn new things and see, you know, just discover new things.* Mrs. Jones however, was more focussed upon the health benefits of physical activity (citing it as an important part of living a balanced life). She was convinced that educating parents was vital, commenting that many know that they should be encouraging more physical activity, but are not sure how to do so.
Both George and Sophie were particularly keen on learning new skills and challenging themselves, and viewed this as motivation for taking part in physical activity promotion. *George: Well because like that new thing could be sort of very attractive and then like if you know lots of activities like you play them more, like if you only knew how to play football you’ll get bored of football after a while.* Both parents agreed that in trying to ‘sell studies’, an understanding of family context is important. For example, already-active children may be attracted to the social element of a research study, whilst those more sedentary might be encouraged by the opportunity to increase physical confidence and competence. When asked about other incentives for research participation, Mrs. Jones dismissed childcare provision and financial reward as insufficient. *Mrs. Jones: … this is not, the ‘provides childcare’ is not terribly important to me…* Both parents stressed the need for researchers to make the measurable benefits of study participation clear, and to provide regular feedback to families. They suggested that without a perceived return on investment (of time, effort, and/or resources), parents would be unwilling to register their children in a study. Additionally, Mr. Jones commented that sustaining interest through continued contact may increase study retention rates.

### Barriers to involvement

Increased time commitment or scheduling difficulties were quoted as the most pertinent barriers to involvement in physical activity research. This was especially true for families with several children;

Mother: boy, 10y: *If two children of different ages had to be in two places at once that would be difficult*

Parents suggested that research activities should be kept outside of both working hours and extra-curricular club periods;

Mother: boy, 10y: *But often school clubs finish at what, 4.30 pm, and because they don’t go to bed ‘til 8.30 pm, there’s a four hour gap and often you and your friends, you’ll be either watching TV, or on your Xbox…when actually if there was something really good to do in a sports centre that was absolutely targeted at your age that would be great*

In contrast, single parents considered that providing intervention activities during working hours (when paid childcare would otherwise be required) would encourage participation. Activities requiring extensive time investment, or attendance at inconvenient times of the day, were poorly received. Some families mentioned challenges with transport, but they noted that these could be overcome if the perceived benefits of involvement were sufficient (“*if there is obvious benefit”* (father: boy, 10y)).

## Box 3 The Smith family

**Table:** 

The Smith family was of lower socio-economic status than the study average and comprised a mother and single child (Tim, aged 9 years). Tim cited great enthusiasm for sport; in particular, he enjoys playing football. The focus group did not capture his full attention, and he was distracted at times (for example, blowing raspberries into the voice recorder). Miss Smith was very engaged in Tim’s development. She was aware of the potential benefits of engaging in physical activity, and ranked improved social skills as a key outcome of interest. Perhaps given the behavioural issues of her son, she was particularly keen on developing positive connections with other families. *Miss Smith: I think improved social skills is good, especially for shy children, I know he didn’t seem it but he can be shy sometimes so that’s quite nice to get them active and sort of connect to other people.* Related to these family constraints, she cited time commitment and scheduling clashes as being key barriers to research participation. She suggested that coordinating her own work, the target child’s time with his father, and paid childcare were difficult – and that adding further scheduled activities would be a challenge. *Miss Smith: Probably time commitment would be a big one for me ‘cos I’m working, Tim’s at school or at the childminders and then he’s at his dad’s and then we struggle to find time to do stuff anyway.* However, the provision of childcare would be an incentive for taking part in a research study, if such time constraints were managed. As a single parent, she suggested that childcare (particularly if there was no cost involved) would be beneficial. *Miss Smith: Yeah, providing childcare would be relevant to me ‘cos, as you know, I’m on my own anyway, so that’s always quite handy to have sort of groups or things that they would go to, yeah.* For Tim, having fun and spending time with friends were of primary importance. During the session, several of his friends attended the family home, asking him to play outside with them. Unsurprisingly, he was not concerned about being healthy, and did not view educational benefits of being sufficiently motivating. Tim did not agree with his mother about being shy; and commented that the only reason he would decline participation in an activity was a lack of interest.
*Tim: Because I’m not really worried or shy or lazy or scared, I’m just not bothered to play it and don’t really like it that much.*

## Discussion

Given the potential benefit of engaging the family in physical activity promotion [1, 2, 13], understanding how best to recruit for, and retain participation in, such intervention research is essential. Work conducted to date has included *either* parents *or* children, but has not involved all members of the family [7–9]. This study was the first to do so, and included parents, children (including siblings), and, in one case, grandparents. Perceived benefits of research participation were explored, and families were able to identify the elements of a physical activity intervention that would encourage their involvement. Challenges or barriers to participation were also debated between family members.

When recruiting, making the potential benefits of research participation clear to families was deemed essential. In particular, parents perceived educational, social, and health outcomes to be most relevant (whilst children cited ‘fun’ as a primary motivation for involvement). Strong advertising materials, which succinctly outline the possible advantages of involvement (whilst still clarifying any possible risks), may be useful in increasing initial expressions of interest. This advice is mirrored in Schoeppe and colleagues’ REACH strategies, who list providing clear and simple information describing expected study benefits, as critical to effective recruitment [6].

This focus group study was innovative in the inclusion of all family members, and the thorough exploration of incentives and barriers to research participation. This study was conceived in response to calls for better understanding of how best to recruit and retain participants in family-based physical activity research [3]. However, as if to further highlight the challenging nature of such work, enrolling families into the present study was difficult (see Fig. 1). The relatively high socio-economic background of participants may limit wider application. Although every effort was made to recruit a range of families, the disproportionate wealth in Cambridge and the surrounding areas made this problematic. Future studies should target participants from low socioeconomic backgrounds to investigate whether there are unique barriers and facilitators to research participation is this ‘hard to reach’ population. To offset this potential source of bias, case studies exploring individual incentives for and barriers to research participation, were also included. The inclusion of such detailed family narrative enables further understanding of the complexity of family context.

Regular feedback on the children’s progress and performance was suggested to retain families in physical activity interventions. Given the importance of motivation in adherence to similar trials [14–20], offering regular feedback may be essential in retaining families in physical activity interventions. Additionally, parents suggested that receiving information about their children’s health and behaviour, which they may not otherwise access, would provide sufficient incentive for engagement in research. However, researchers need to be mindful that intervention efficacy is often contingent upon participants’ understanding of their own behaviour. Ensuring that inactive families are aware of their inactivity, through effective feedback strategies, may increase their motivation to change behavior, and subsequent behavior [21]. This may however have unintended consequences for an evaluation, including positive behavior change in the control group, which may lead to an inability to observe true intervention effects [22].

Single parent families may be a key target group for physical activity intervention. Observational evidence suggests that family structure is important in the physical activity levels of children; for example, girls from single parent families have reported significantly more minutes per day watching television compared with girls from two-parent families [23]. In the present study, the data provided from single parent families differed considerably from that provided by multiple-parent families, or families in which childcare was available from other relatives. Single parents saw the provision of childcare alone as a sufficient incentive for involvement; particularly, offering an intervention during working hours (when paid childcare would otherwise be required) would encourage participation. When recruiting, researchers should consider family structure, and tailor advertising materials and intervention delivery to suit the target context.

## Conclusion

These key lessons may contribute to the development of effective recruitment and retention strategies for children and their families. Making benefits clear to families, providing regular feedback, and carefully considering family structure, may prove useful in achieving desired research participation. This may subsequently assist in engaging families in interventions to increase physical activity in children

## References

[1] Sallis JF, Prochaska JJ, Taylor WC (2000). A review of correlates of physical activity of children and adolescents. Med Sci Sport Exerc.

[2] Van Sluijs EMF, McMinn AM, Griffin SJ (2007). Effectiveness of interventions to promote physical activity in children and adolescents: systematic review of controlled trials. BMJ.

[3] O’Connor TM, Jago R, Baranowski T (2009). Engaging parents to increase youth physical activity a systematic review. Am J Prev Med.

[4] Kriemler S, Meyer U, Martin E, van Sluijs EMF, Andersen LB, Martin BW (2011). Effect of school-based interventions on physical activity and fitness in children and adolescents: a review of reviews and systematic update. Br J Sports Med.

[5] Karlson CW, Rapoff MA (2009). Attrition in randomized controlled trials for pediatric chronic conditions. J Pediatr Psychol.

[6] Schoeppe S, Oliver M, Badland HM, Burke M, Duncan MJ (2014). Recruitment and retention of children in behavioral health risk factor studies: REACH strategies. Int J Behav Med.

[7] Bentley GF, Goodred JK, Jago R, Sebire SJ, Lucas PJ, Fox KR (2012). Parents’ views on child physical activity and their implications for physical activity parenting interventions: a qualitative study. BMC Pediatr.

[8] Jago R, Steeds JK, Bentley GF, Sebire SJ, Lucas PJ, Fox KR (2012). Designing a physical activity parenting course: parental views on recruitment, content and delivery. BMC Public Health.

[9] Davison KK, Jurkowski JM, Li K, Kranz S, Lawson HA (2013). A childhood obesity intervention developed by families for families: results from a pilot study. Int J Behav Nutr Phys Act.

[10] Sahlqvist S, Song Y, Bull F, Adams E, Preston J, Ogilvie D (2011). Effect of questionnaire length, personalisation and reminder type on response rate to a complex postal survey: randomised controlled trial. BMC Med Res Methodol.

[11] Statistics N (2007). Neighbourhood summary deprivation tab.

[12] Attride-Stirling J (2001). Thematic networks: an analytic tool for qualitative research. Qual Res.

[13] Brown HE, Atkin AJ, Panter J, Wong G, Chinapaw MJ, van Sluijs EMF. Family-based interventions to increase physical activity in children: a systematic review, meta-analysis and realist synthesis. Under Rev. (in press).

[14] Teixeira PJ, Going SB, Houtkooper LB, Cussler EC, Metcalfe LL, Blew RM (2004). Pretreatment predictors of attrition and successful weight management in women. Int J Obes Relat Metab Disord.

[15] Jeffery RW, Bjornson-Benson WM, Rosenthal BS, Lindquist RA, Kurth CL, Johnson SL (1984). Correlates of weight loss and its maintenance over two years of follow-up among middle-aged men. Prev Med (Baltim).

[16] Stevens VJ, Rossner J, Greenlick M, Stevens N, Frankel HM, Craddick S (1989). Freedom from fat: a contemporary multi-component weight loss program for the general population of obese adults. J Am Diet Assoc.

[17] Streit KJ, Stevens NH, Stevens VJ, Rossner J (1991). Food records: a predictor and modifier of weight change in a long-term weight loss program. J Am Diet Assoc.

[18] Williams GC, Grow VM, Freedman ZR, Ryan RM, Deci EL (1996). Motivational predictors of weight loss and weight-loss maintenance. J Pers Soc Psychol.

[19] Chao D, Farmer DF, Sevick MA, Espeland MA, Vitolins M, Naughton MJ (2000). The value of session attendance in a weight-loss intervention. Am J Health Behav.

[20] Wilcox S, Shumaker SA, Bowen DJ, Naughton MJ, Rosal MC, Ludlam SE (2001). Promoting adherence and retention to clinical trials in special populations: a Women’s health initiative workshop. Control Clin Trials.

[21] Lubans DR, Morgan PJ, Tudor-Locke C (2009). A systematic review of studies using pedometers to promote physical activity among youth. Prev Med.

[22] Van Sluijs EMF, van Poppel MNM, Twisk JWR, van Mechelen W (2006). Physical activity measurements affected participants’ behavior in a randomized controlled trial. J Clin Epidemiol.

[23] Bagley S, Salmon J, Crawford D (2006). Family structure and children’s television viewing and physical activity. Med Sci Sports Exerc.

